# Case report: Ketogenic diet alleviated anxiety and depression associated with insulin-dependent diabetes management

**DOI:** 10.3389/fnut.2024.1404842

**Published:** 2024-10-30

**Authors:** Eirin Winje, Ian Lake, Simon N. Dankel

**Affiliations:** ^1^Independent Researcher, Drøbak, Norway; ^2^General Practitioner NHS, Gloucestershire, United Kingdom; ^3^Department of Clinical Science, University of Bergen, Bergen, Norway

**Keywords:** ketogenic diet, type 1 diabetes mellitus, diabetes distress, anxiety, depression, glycogen depletion, ketoacidosis, eating disorders

## Abstract

Differentiating between an irrational versus a rational fear of hypoglycemia has treatment implications and presents significant challenge for clinicians facing patients with type 1 diabetes, illustrated in this case. A 39-year-old woman with autoimmune-positive insulin-dependent diabetes sought help to alleviate severe diabetes distress, and symptoms of depression and anxiety, associated with unpredictable drastic blood glucose drops. After exhausting conventional methods, she adopted a ketogenic diet (KD). Her glucose values decreased from around 20 mmol/L to 12 mmol/L (360 mg/dL to 216 mg/dL) in the first days. Then, by combining a KD with an insulin pump, her time in optimal glucose range increased from 8 to 51% after 2 months, reducing her HbA1c with 25 mmol/mol (2.2%). This reduced biological and psychological stress, immediately improving her mental health and renewing her hope for the future. The main concerns regarding KD in patients with comorbid type 1 diabetes is the assumed increased risk of ketoacidosis, theoretical depletion of glycogen stores, and a potential adverse effect of saturated fat on cardiovascular risk factors. These concerns are evaluated against existing empirical evidence, suggesting instead that a KD may protect against acidosis, hypoglycemia, and cardiovascular risk. The present case, together with available data, indicate that patients with type 1 diabetes experiencing high levels of biological and psychological stress should be informed of the expected benefits and possible risks associated with a KD, to ensure their right to take informed decisions regarding their diabetes management.

## 1 Introduction

Although the hallmark of type 1 diabetes is the autoimmune destruction of insulin producing *β*-cells, necessitating exogenous insulin, living with diabetes comes with a high psychological burden. This “diabetes distress” can include feelings of being overwhelmed, powerless and hopeless, along with fears of acute and long-term consequences. It includes the stress and frustration of daily management and conflicts with family and health care providers (1, 2). This can develop into a vicious cycle of increasing distress leading to emotional numbing and deteriorated glycemic control, which then in turn increases the psychological burden (3). Approximately 40–60% of individuals with type 1 diabetes experience moderate to high levels of this distress (4). This group is especially at risk for “diabetes burnout”; a state characterized by feeling exhausted, seen in combination with reduced self-care and diabetes management (2). These symptoms may be intense enough to fulfill diagnostic criteria for anxiety and depression. Individuals with type 1 diabetes are believed to have at least double the risk of various mental disorders and even suicide (5–7).

The situation is further complicated as about 50% of people with type 1 diabetes develop insulin resistance (8), worsening prognosis. To further complicate the picture; insulin resistance in the blood–brain barrier can occur without whole body insulin resistance, making it harder to detect, but with potential harm to the brain (9, 10). Alarmingly, there is an estimated loss of 100 days of life for each year a person’s HbA1c exceeds 58 mmol/mol (7.5%) (11).

Insulin resistance, and high levels of insulin and glucose are associated with mitochondrial dysfunction, neuroinflammation, neurotransmitter imbalances, glucose hypometabolism, and lower volume in parts of the brain (5, 9, 12, 13), all of which are also associated with mental disorders (10, 14–16).

Numerous factors complicate the estimation of appropriate insulin dosage. Two key factors are the variability in the effect of insulin administered subcutaneously (17), and miscalculations of the carbohydrate content in food, which may lead to a mismatch with the estimated insulin dosage (18). For example, assuming a combined error of 25%, the absolute value of the error will be lower with lower insulin doses. For instance, a 25% error at a “true” carbohydrate intake of 80 g will result in a miscalculation of 20 g in either direction. In contrast, a “true” carbohydrate intake of 10 g will result in a miscalculation of just 2.5 g in either direction. An insulin pump might compensate for the 2.5 g miscalculation, while a miscalculation of 20 g will significantly interfere with daily life.

Studies of various designs show that a low-carbohydrate diet (under about 100 g/day) lowers HbA1c, even approaching the prediabetic values of <48 mmol/mol (<6.5%), reduces glucose variability, and time spent in hypoglycemia without reducing quality of life (19–23). The ketogenic diet (about 20- 50 g/day of carbohydrates) appears even more beneficial, with HbA1c levels approaching non-diabetic values <42 mmol/mol (<6.0%) and normalize weigh (24–32). In addition, nutritional ketosis protects against the potentially detrimental effects of insulin resistance, high levels of insulin, and high/variable levels of glucose, thereby probably preventing and even treating symptoms of psychiatric disease and neurodegeneration (33). See overviews of the proposed mechanisms elsewhere, for example (34–38).

A key contributor to reduced mental health is the unpredictable nature of type 1 diabetes. A typical experience is that glucose levels tend to drop significantly during even light physical activity and exercise. This is likely due to heightened insulin mediated activation of the signaling pathway controlling GLUT4 glucose transporter activity (39).This effect may be amplified by (i) absence of the glucose sparing effect of nutritional ketosis (40, 41), and (ii) the slightly higher insulin levels by subcutaneous injected insulin compared to the release of endogenous insulin by the pancreas into the portal vein (17). Insulin levels can therefore often become high enough to cause uncomfortable drops during normal daily activities like house cleaning or short walks. This might increase diabetes distress and induce a fear of the very real threat associated with hypoglycemia. However, most cases of hypoglycemia can be easily corrected, often resulting in different threat assessments between healthcare personnel and their patients. Therefore, distinguishing between an anxiety and a rational fear of hypoglycemia has treatment implications, but may be challenging in clinical practice.

This case-report illustrates how introducing a KD mitigated intense diabetes distress and even symptoms of severe anxiety and depression.

## 2 Case description

### 2.1 Patient information and evaluation

A 39-year-old woman with a history of trauma and autoimmune-positive insulin-dependent diabetes for about 2 years was referred to an experienced licensed clinical psychologist familiar with diabetes treatment. Past severe and unpredictable hypoglycemia had frightened the patient, and as a single mother of two, she was particularly concerned about dangerous hypoglycemia. Despite numerous interventions from several diabetes teams, including psychological and dietary approaches, unpredictable glucose levels remained a threat. The expected results did not materialize when she intervened as instructed, leading to increased frustration and fear. Consequently, she felt safe only at high glucose levels (15–20 mmol/L; 270–360 mg/dL). She felt blamed for her fear and failure to control her diabetes. Increasingly desperate, she began to accept the prospect of rapidly deteriorating health and death within a few years, even making plans for her children’s care after her death. She reluctantly agreed to start an insulin pump but was terrified of having more insulin and being dependent on a machine.

An emergency session was arranged on a Tuesday as she was scheduled to commence pump treatment on the following Friday.

Based on the patient’s descriptions in the clinical assessment interview, the psychologist concluded that the patient’s psychological pain was severe, with high levels of diabetes distress, approaching panic. This distress fulfilled the diagnostic criteria for F 40.2 and F 32; specific phobia and depression. Her anxiety symptoms included intense fear of hypoglycemia and insulin, causing her to omit and avoid necessary insulin doses. She felt trapped and a loss of control, becoming agitated and autonomically hyperactivated. Her depression was marked of by a sense of surrender to the prospect of dying due to her diabetes, alongside a loss of energy, interest and joy. She experienced negative thoughts about the future, feelings of guilt, unworthiness, and cognitive difficulties such as problems with concentration, attention and memory. These symptoms had persisted for at least 1 year, had rendered her unable to work and limited her social life. In combination with the fatigue caused by the hyperglycemia, she could barely manage to take care of herself and her children. These symptoms are closely related to her life with diabetes, and most likely exacerbated by hyperglycemia and the unpredictable and uncontrollable glucose drops.

By accepting the patient’s narrative, the logical course of action appeared to be to minimize any unnecessary insulin requirement due to “normal” carbohydrate consumption and harness the advantages of a ketogenic diet (KD). The current KD was defined as a diet allowing her body to produce ketones.

### 2.2 Intervention

Due to the patient’s severe pain and time constraints, the psychologist promptly provided information on how a KD could mitigate sudden glucose drops, thus addressing psychological distress and biological stress. The psychoeducation included possible benefits and risks of utilizing a KD for mental health with comorbid diabetes, and practical tips on how to implement it, allowing for individualization of the diet. Generally, important aspects of a KD to consider for insulin-dependent diabetes includes:

1. Ensure sufficient insulin: Use a continuous glucose monitor (CGM) with “high alarm” at 10–12 mmol/L (180–215 mg/dL). Consider adding insulin if glucose values exceed 10 mmol/L (180 mg/dL). Expect decreased insulin need of up to 50%. Bolus for 5-10 g carbohydrates when eating a keto meal, to cover protein and notify the pump of the meal. Always have an extra insulin pen handy, in case of pump malfunctions.
2. Prevent and rescue hypoglycemia: The CGM should alert when glucose falls below 4 mmol/L (72 mg/dL). Consider consuming 2-5 g glucose if the alarm sounds, preferably in tablet form. This prevents overeating and subsequent hyperglycemia.
3. Enter nutritional ketosis: Base food intake decisions on three guidelines: (i) Prioritize protein: include meat, fish, egg in all/most meals. (ii) Reduce carbohydrates: avoid sugar, starch, rice, potatoes, bread, and pasta. Aim for 25-50 g carbohydrate per day. Foods with less than 5% carbohydrate per 100 g are typically acceptable. (iii) Add fat to satiety and taste: feel free to include saturated fat, but limit highly processed vegetable oils.
4. Monitor ketones: Aim for blood ketones between 0.5- 3 mmol/L (42). Initially measure a few times a week, then two times or less a month may suffice. If ketone levels are 3 mmol/L or higher, consume about 10-30 g of carbohydrates and administer matched insulin. Test ketone levels if you get sick, unusually thirsty, headache, nausea, stomachache, or the like.
5. Regarding other medication: Notify relevant healthcare personnel of the change in diet. Do not combine with SGLT-2 inhibitor. Regularly assess the need to adjust blood pressure medication and psychopharmaceuticals.

## 3 Results

The patient embraced the KD after her first session on the Tuesday, choosing food based on the third guideline above. By the following Thursday, she reported a steady decline in her glucose from about 20 mmol/L to 12 mmol/L (360 to 216 mg/dL). She reported feeling safe for the first time in years and a renewed hope for the future, even her children noticed a positive change in her during these first few days on the new diet. This signified a relief in her depressive state and a reduced threat assessment, including her autonomic overactivation, allowing her to start the hybrid closed-loop insulin pump the following Friday. Thus, this had mitigated her avoidance to insulin, relieved her state of panic and severe depression.

The following months, the combination of her reduced insulin need and the insulin pump, her glucose levels significantly stabilized, with increased time in range [glucose values between 4 and 10 mmol/L (72–180 mg/dL)] from 3 to 51%, reducing her HbA1c from 83 to 58 mmol/mol (9.7 to 7.5%) (See Table 1).

**Table 1:** Information from continuous glucose monitor.

Time spent in:	T1 16th September to 29th September 2023	T2 20th November to 4th December 2023
Hypoglycemia % 3–3.8 mmol/L (54–64.4 mg/dL)	0	0
Aim % 3.9–10 mmol/L (70.2–180 mg/dL)	3	51
Hyperglycemia % 10.1–13.9 mmol/L (181.8–250.2 mg/dL)	19	49
Hyperglycemia % >13,9 mmol/L (>250.2 mg/dL)	78	0
Mean glucose mmol/L (mg/dL)	16.7 (300.6)	9.9 (178.2)
Standard deviation	No info	1.2
Coefficient of variation %	22.1	12.5
HbA1c mmol/mol (%)	83 (9.7)	58 (7.5)

T1 is the14 days before starting the ketogenic diet, the insulin pump and a HbA1c test. T2 is the 14 days before the second HbA1c test, about 2 months after T1.

From 2 days after the emergency session and onwards, the clinician observed the patient’s amazement and joy of her reduced glucose variability, her relief of being believed in her motivation and efforts to manage her diabetes, and a clinically significant reduction in fear and depression.

## 4 Discussion

Although fulfilling formal criteria for depression and anxiety, the patient’s mental state and behavior was interpreted as logical reactions to a dangerous situation, not as irrational fear or poor coping. By addressing the diet, the psychological and biological stress on the brain could be reduced.

Despite the promising effects on diabetes management and mental health outlined in the introduction, both regulatory bodies and the low-carbohydrate community have urged caution when considering nutritional ketosis for people with diabetes. There are three main concerns delaying the implementation of the KD in clinical practice, all of which can be mitigated by evidence. Addressing these concerns is crucial to ensure that patients receive the information they need to make informed dietary choices. Had the current patient received this information earlier, she may not have developed her anxiety and depression, which lead to her avoidance of pump treatment.

### 4.1 Diabetic ketoacidosis

Diabetic Ketoacidosis (DKA) is a potentially lethal condition marked by hyperglycemia [serum glucose >13.9 mmol/L (>250 mg/dL)], increased anion cap metabolic acidosis (anion gap >10–12, serum bicarbonate <18 mEq/L and/or pH <7.3), and ketosis (> 2 mmol/L) (43). Usually, DKA is seen in combination with insulin deficiency and/or increased amounts of counter-regulatory hormones like catecholamines, glucagon, cortisol, and growth hormone (44). The acidosis has been attributed to the increased level of ketones (43). However, the ketones acetoacetate and beta-hydroxybutyrate are not produced as acids but as conjugate bases, and acetone is neither an acid nor a base (45). The acidosis can therefore not be directly caused by the presence of ketone bodies. The acidosis is more likely a related consequence linked to the Krebs cycle’s maximum oxidation rate of Acetyl-CoA. Further lipolysis after the maximum oxidation is reached, will result in the release of a substantial number of protons per triglyceride molecule. And when these are not consumed by complex 1 in the electron transport chain, acidification may occur (45).

In contrast to DKA, in nutritional ketosis the blood pH remains withing normal limits and, glucose values are normal, but ketones are about 0.5–3 mmol/L (40, 46). Surprisingly, keto-adaptation has been observed to enhance the oxidation rate in ultra-athletes (47, 48). This could mean that the acidotic process associated with ketone production may be deferred through a KD, and possibly further postponed by including exercise (41, 46, 49).

Information is scarce on the incidence, prevalence, and risk factors of acidosis in type 1 diabetes patients in nutritional ketosis. One study reported a 1% incidence (24), compared to an estimated annual 5–8% incidence in the broader type 1 diabetes population (not on KD) (44). This information is relevant for this case, as it means that the patient is actually safer from a DKA on a KD.

In our opinion, a more pressing concern for type 1 diabetes patients is an increased risk of DKA during acute infections or pump malfunctions. However, if a pump with fast acting insulin malfunctions, the body’s insulin reserve depletes within hours. It is unclear how much ketones, induced by nutritional ketosis, will impact DKA development, as it is not the ketones themselves that are acidic. More research is needed. In addition, near normal glucose levels decreases tolerance for glucose fluctuations, thereby rapidly alerting the individual if insulin is needed due to hyperglycemia. And importantly, the improved predictability by combining KD with an insulin pump significantly reduces the mental burden related to diabetes, which was exactly what the current patient needed to reduce her anxiety and depression.

Euglycemic DKA involves acidosis with normal glucose levels and accounts for about 2.6–7% of known DKA cases (43). The main contributor appears to be the off-label use of SGLT-2 inhibitors. Other risk factors could include low-fat zero-carb diets, combined with prolonged fasting and intense exercise (50). Thus, the main contributor is not a KD, which is a high-fat low-carb diet that encourages the individual to eat when hungry and match insulin to maintain normal glucose levels. Keto-adapted people with type 1 diabetes often experience increased energy, satiety, and can thrive during intensive multi-day exercise, even when fasted (31, 32). As the current patient was not on SGLT-2 inhibitors but adhered to the KD aiming to better match her insulin injections to her need, the risk of euglycemic DKA was low.

Regardless of their diet and treatment, all individuals with type 1 diabetes must be vigilant about the risk of DKA.

### 4.2 Hypoglycemia and empty glycogen stores?

Intensive insulin treatment, leading to lower HbA1c levels, has been associated with a threefold increase in hypoglycemia frequency (51). Transitioning to a KD significantly reduces insulin needs, potentially causing a slight increase in hypoglycemic episodes, indicating that further insulin reductions are necessary. As the current patient suffered from severe hyperglycemia, the clinician calculated that the reduced insulin need due to KD would resolve the hyperglycemia without triggering anxiety, avoidance of insulin, nor lead to hypoglycemia. The insulin levels the patient managed to inject would be sufficient.

A primary concern other clinicians has is the belief that a KD depletes glycogen stores before ketone production occurs, potentially rendering glucagon injections ineffective during severe hypoglycemia. A small study (n = 10) found that glucagon injection resulted in a higher glucose rise after a high-carb week compared to a low-carb week. However, the rise was sufficient to rescue hypoglycemia in both groups (52), thereby rendering this finding irrelevant.

Three counterpoints to the glycogen storage total “depletion” notion include: (i) Similar levels of resting muscle glycogen stores and glycogen depletion after 180 min running were observed in keto-adapted endurance athletes and athletes consuming “high-carbohydrate” diet (49), potentially due to increased gluconeogenesis rates and a glucose-sparing effect of ketones (41, 46). (ii) During physical activity, muscles primarily use fat for fuel, and increased gluconeogenesis rates contribute to stabilize blood glucose (41). (iii) If hypoglycemia occurs, the brain uses ketones for fuel, mitigating the impact of reduced glucose availability in keto-adapted individuals (32, 53).

By informing the current patient of the benefits of becoming keto-adapted, including the additional fuel for the brain, the patient was able to accept the prospect of more insulin on board delivered by the upcoming pump treatment.

### 4.3 Cardiovascular disease (CVD)

CVD is the leading cause of death in type 1 diabetes patients (54). The main concern is that a KD, often relatively high in saturated fat, can increase low-density lipoprotein cholesterol (LDL-C) and therefore presumably the risk of CVD. However, there are five counter arguments: (i) Individuals with lower levels of LDL-C can become just as atherosclerotic as people with high LDL-C, if not more (55–57). (ii) A recent study found LDL-C not to be a significant risk factor in type 1 diabetes patients (58), suggesting that interventions should not be based solely on theory, but tested in the relevant populations. (iii) The understanding of CVD is evolving, now attributing more atherosclerotic properties to insulin resistance, inflammation and a subgroup of LDL particles, e.g., small dense LDLs (59, 60). (iv) A KD has been found to redistribute the fractions of small dense and big fluffy LDL, to lower circulating triacylglycerols, and to reduce other more important risk factors associated with high glucose levels and insulin resistance (59, 61). (v) The heart might benefit from running on ketones (62). Furthermore, patients with a normal BMI might be more likely to develop a lean-mass hyper-responder phenotype (LMHR) of very high LDL in combination with high HDL and low triacylglycerols (61), related to the carbohydrate restriction rather than the high saturated fat intake (63). This phenotype may not promote CVD risk in the same way as normal dyslipidemia, and there is therefore consensus that CVD risk assessment needs to be individualized according to metabolic state and other factors (64).The CVD risk was a not a current concern for this patient. In her state, her immediate priority was finding relief to take care of her children. Her main goal was to overcome her fear of insulin and resolve the toxic hyperglycemia. However, her HDL, triacylglycerol, and LDL levels did not indicate signs of LMHR, but her values will be closely monitored.

### 4.4 Strength and weaknesses

This report illustrates how a KD can be used in a normal clinical setting. A weakness is a lack of assessment scales for diabetes distress, anxiety, and depression pre and post the KD intervention, and a relatively short follow-up time.

## 5 Take-away lessons

Insulin avoidance due to anxiety and depression may be relieved through a KD. This observation supports the emerging view that a KD is a viable option for some individuals with type 1 diabetes. Despite concerns about KD for individuals with type 1 diabetes, there exist counterarguments and contradictory evidence, suggesting that the perceived risks might not be as significant as initially thought. On the contrary, being keto-adapted might protect against DKA, hypoglycemia, and CVD, in addition to improve mental health both through biological and psychological mechanisms.

Patients struggling to live with diabetes should be provided with comprehensive information about potential benefits and risks of adopting a KD, to ensure their right to make informed treatment decisions, including dietary interventions.

## 6 Patient perspective

Pre KD: In the summer of 2023, I was severely ill, overwhelmingly fatigued and could no longer function properly. Often, I was haunted by the thought that I may not have much time left and filled with despair at the thought of not being there for my children. I began to plan for their care and well-being if I were to die soon. Consequently, I realized that my only option was to try an insulin pump, a prospect that filled me with dread. I got an emergency phone appointment with a new psychologist. During the call, I was so affected by hyperglycemia that I struggled to think clearly and concentrate. I shared my challenges with managing diabetes and my fear about starting the insulin pump. I immediately felt understood and got quickly reassured by the psychologist’s guidance on initiating insulin pump therapy and transitioning to a KD.

On a KD: In a remarkably short period, I noticed significant improvements. With each passing day, I felt I was regaining my vitality. Thanks to the guidance and support of my psychologist, I have been immersing myself in learning about diabetes and KD, gaining new knowledge every day. The patient has continued the KD for 1 year and is closely followed up by her diabetes team.

## Data Availability

The original contributions presented in this paper are based on the patient’s medical records, which are not publicly available due to privacy restrictions. Inquiries can be directed to the corresponding author.

## References

[1] Fisher L Polonsky WH Hessler D. Addressing diabetes distress in clinical care: a practical guide. Diabet Med. (2019) 36:803–12. doi: 10.1111/dme.13967 30985025

[2] Kiriella DA Islam S Oridota O Sohler N Dessenne C de Beaufort C. Unraveling the concepts of distress, burnout, and depression in type 1 diabetes: a scoping review. EClinicalMedicine. (2021) 40:101118. doi: 10.1016/j.eclinm.2021.101118, PMID: 34485879 PMC8408521

[3] Duinkerken E Snoek FJ Wit M. The cognitive and psychological effects of living with type 1 diabetes: a narrative review. Diabet Med. (2020) 37:555–63. doi: 10.1111/dme.14216, PMID: 31850538 PMC7154747

[4] Hedge V Carter K Downey W Sharp H. Prevalence of diabetes distress among adolescents with type 1 diabetes mellitus. J Nurse Pract. (2023) 19:104383. doi: 10.1016/j.nurpra.2022.06.008

[5] Martin H Bullich S Guiard BP Fioramonti X. The impact of insulin on the serotonergic system and consequences on diabetes-associated mood disorders. J Neuroendocrinol. (2021) 33:e12928. doi: 10.1111/jne.12928 33506507

[6] Benton M Cleal B Prina M Baykoca J Willaing I Price H. Prevalence of mental disorders in people living with type 1 diabetes: a systematic literature review and meta-analysis. Gen Hosp Psychiatry. (2023) 80:1–16. doi: 10.1016/j.genhosppsych.2022.11.004 36493531

[7] Wilhelmsen-Langeland A Handelsby N Wisting L Winje E. Diabetes type 1 øker risiko for selvmord: Hva kan psykologen gjøre? Tidsskrift Norsk psykologforening. (2024) 61:90–9. doi: 10.52734/SNXA8325

[8] Kietsiriroje N Pearson S Campbell M Ariens RAS Ajjan RA. Double diabetes: a distinct high-risk group? Diabetes Obes Metab. (2019) 21:2609–18. doi: 10.1111/dom.13848 31373146

[9] Milstein JL Ferris HA. The brain as an insulin-sensitive metabolic organ. Mol Metab. (2021) 52:101234. doi: 10.1016/j.molmet.2021.101234, PMID: 33845179 PMC8513144

[10] Sullivan M Fernandez-Aranda F Camacho-Barcia L Harkin A Macrì S Mora-Maltas B. Insulin and disorders of behavioural flexibility. Neurosci Biobehav Rev. (2023) 150:105169. doi: 10.1016/j.neubiorev.2023.105169 37059405

[11] Heald AH Stedman M Davies M Livingston M Alshames R Lunt M. Estimating life years lost to diabetes: outcomes from analysis of National Diabetes Audit and Office of National Statistics data. Cardiovasc Endocrinol Metab. (2020) 9:183–5. doi: 10.1097/XCE.0000000000000210, PMID: 33225235 PMC7673790

[12] Neyman O Hershey T. 117Type 1 and type 2 diabetes and the brain In: Alosco Michael L Stern Robert A, editors. The Oxford handbook of adult cognitive disorders. Oxford: Oxford University Press (2019)

[13] Filip P Canna A Moheet A Bednarik P Grohn H Li X. Structural alterations in deep brain structures in type 1 diabetes. Diabetes. (2020) 69:2458–66. doi: 10.2337/db19-1100, PMID: 32839347 PMC7576566

[14] Norwitz NG Dalai SS Palmer CM. Ketogenic diet as a metabolic treatment for mental illness. Curr Opin Endocrinol Diabetes Obes. (2020) 27:269–74. doi: 10.1097/MED.0000000000000564 32773571

[15] Sethi S Ford JM. The role of ketogenic metabolic therapy on the brain in serious mental illness. Rev J Psychiatr Brain Sci. (2022) 7. doi: 10.20900/jpbs.20220009 PMC9728807 36483840

[16] Calkin CV. Insulin resistance takes center stage: a new paradigm in the progression of bipolar disorder. Ann Med. (2019) 51:281–93. doi: 10.1080/07853890.2019.1659511, PMID: 31453713 PMC7877881

[17] Gradel AKJ Porsgaard T Lykkesfeldt J Seested T Gram-Nielsen S Kristensen NR. Factors affecting the absorption of subcutaneously administered insulin: effect on variability. J Diabetes Res. (2018) 2018:1–17. doi: 10.1155/2018/1205121 PMC6079517 30116732

[18] Kawamura T Takamura C Hirose M Hashimoto T Higashide T Kashihara Y. The factors affecting on estimation of carbohydrate content of meals in carbohydrate counting. Clin Pediatr Endocrinol. (2015) 24:153–65. doi: 10.1297/cpe.24.153, PMID: 26568656 PMC4628950

[19] Nielsen JV Jönsson E Ivarsson A. A low carbohydrate diet in type 1 diabetes. Ups J Med Sci. (2005) 110:267–73. doi: 10.3109/2000-1967-074 16454166

[20] Nielsen JV Gando C Joensson E Paulsson C. Low carbohydrate diet in type 1 diabetes, long-term improvement and adherence: a clinical audit. Diabetol Metab Syndr. (2012) 4:23. doi: 10.1186/1758-5996-4-23, PMID: 22650646 PMC3583262

[21] Schmidt S Christensen MB Serifovski N Damm-Frydenberg C Jensen JB Floyel T. Low versus high carbohydrate diet in type 1 diabetes: a 12-week randomized open-label crossover study. Diabetes Obes Metab. (2019) 21:1680–8. doi: 10.1111/dom.13725 30924570

[22] Krebs JD Strong AP Cresswell P Reynolds AN Hanna A Haeusler S. A randomised trial of the feasibility of a low carbohydrate diet vs standard carbohydrate counting in adults with type 1 diabetes taking body weight into account. Asia Pac J Clin Nutr. (2016) 25:78–84. doi: 10.6133/apjcn.2016.25.1.11 26965765

[23] Turton JL Brinkworth GD Parker HM Lim D Lee K Rush A. Effects of a low-carbohydrate diet in adults with type 1 diabetes management: a single arm non-randomised clinical trial. PLoS One. (2023) 18:e0288440. doi: 10.1371/journal.pone.0288440, PMID: 37432920 PMC10335683

[24] Lennerz BDB Barton A Bernstein RK Dikeman RD Diulus C Hallberg S. Management of type1 diabetes with a very low-carbohydrate diet. Pediatrics. (2018) 141:e20173349. doi: 10.1542/peds.2017-3349, PMID: 29735574 PMC6034614

[25] Leow ZZX Guelfi KJ Davis EA Jones TW Fournier PA. The glycaemic benefits of a very-low-carbohydrate ketogenic diet in adults with type 1 diabetes mellitus may be opposed by increased hypoglycaemia risk and dyslipidaemia. Diabet Med. (2018) 35:1258–63. doi: 10.1111/dme.13663 29737587

[26] O'Neill DF Westman EC Bernstein RK. The effects of a low-carbohydrate regimen on glycemic control and serum lipids in diabetes mellitus. Metab Syndr Relat Disord. (2003) 1:291–8. doi: 10.1089/1540419031361345 18370654

[27] Vernon MC Mavropoulos J Transue M Yancy WS Jr Westman EC. Clinical experience of a carbohydrate-restricted diet: effect on diabetes mellitus. Metab Syndr Relat Disord. (2003) 1:233–7. doi: 10.1089/154041903322716714 18370667

[28] Gardemann C Knowles S Marquardt T. Managing type 1 diabetes mellitus with a ketogenic diet. Endocrinol Diabetes Metab Case Rep. (2023) 2023:1–7. doi: 10.1530/EDM-23-0008 PMC10448543 37584373

[29] Buehler LA Noe D Knapp S Isaacs D Pantalone KM. Ketogenic diets in the management of type 1 diabetes: safe or safety concern? Cleve Clin J Med. (2021) 88:547–55. doi: 10.3949/ccjm.88a.20121 34598919

[30] Ranjan A Schmidt S Damm-Frydenberg C Holst JJ Madsbad S Nørgaard K. Short-term effects of a low carbohydrate diet on glycaemic variables and cardiovascular risk markers in patients with type 1 diabetes: a randomized open-label crossover trial. Diabetes Obes Metab. (2017) 19:1479–84. doi: 10.1111/dom.12953 28345762

[31] Nolan J Rush A Kaye J. Glycaemic stability of a cyclist with type 1 diabetes: 4011 km in 20 days on a ketogenic diet. Diabet Med. (2019) 36:1503–7. doi: 10.1111/dme.14049 31197870

[32] Lake I. Nutritional ketosis is well-tolerated, even in type 1 diabetes: the ZeroFive100 project; a proof-of-concept study. Curr Opin Endocrinol Diabetes Obes. (2021) 28:453–62. doi: 10.1097/MED.0000000000000666 34334612

[33] Chung JY Kim OY Song J. Role of ketone bodies in diabetes-induced dementia: sirtuins, insulin resistance, synaptic plasticity, mitochondrial dysfunction, and neurotransmitter. Nutr Rev. (2022) 80:774–85. doi: 10.1093/nutrit/nuab118, PMID: 34957519 PMC8907488

[34] Phillips MCL. Fasting as a therapy in neurological disease. Nutrients. (2019) 11:2501. doi: 10.3390/nu11102501, PMID: 31627405 PMC6836141

[35] Kovacs Z D'Agostino DP Diamond D Kindy MS Rogers C Ari C. Therapeutic potential of exogenous ketone supplement induced ketosis in the treatment of psychiatric disorders: review of current literature. Front Psych. (2019) 10:363. doi: 10.3389/fpsyt.2019.00363, PMID: 31178772 PMC6543248

[36] Morris G Maes M Berk M Carvalho AF Puri BK. Nutritional ketosis as an intervention to relieve astrogliosis: possible therapeutic applications in the treatment of neurodegenerative and neuroprogressive disorders. Eur Psychiatry. (2020) 63:e8. doi: 10.1192/j.eurpsy.2019.13 32093791 PMC8057392

[37] Morris G Puri BK Carvalho A Maes M Berk M Ruusunen A. Induced ketosis as a treatment for Neuroprogressive disorders: food for thought? Int J Neuropsychopharmacol. (2020) 23:366–84. doi: 10.1093/ijnp/pyaa008, PMID: 32034911 PMC7311648

[38] Newman JC Verdin E. β-Hydroxybutyrate: much more than a metabolite. Diabetes Res Clin Pract. (2014) 106:173–81. doi: 10.1016/j.diabres.2014.08.009, PMID: 25193333 PMC4414487

[39] Bird SR Hawley JA. Update on the effects of physical activity on insulin sensitivity in humans. BMJ Open Sport Exerc Med. (2017) 2:e000143. doi: 10.1136/bmjsem-2016-000143, PMID: 28879026 PMC5569266

[40] Manninen AH. Metabolic effects of the very-low-carbohydrate diets: misunderstood "villains" of human metabolism. J Int Soc Sports Nutr. (2004) 1:7–11. doi: 10.1186/1550-2783-1-2-7, PMID: 18500949 PMC2129159

[41] Evans M Cogan KE Egan B. Metabolism of ketone bodies during exercise and training: physiological basis for exogenous supplementation. J Physiol. (2017) 595:2857–71. doi: 10.1113/JP273185, PMID: 27861911 PMC5407977

[42] Volek JS Phinney S. Low carbohydrate living. New York, USA: Beyond Obesity. (2011). 1–197.

[43] Dagdeviren M Akkan T Ertugrul DT. Re-emergence of a forgotten diabetes complication: Euglycemic diabetic ketoacidosis. Turk J Emerg Med. (2024) 24:1–7. doi: 10.4103/tjem.tjem_110_23, PMID: 38343522 PMC10852133

[44] Virdi N Poon Y Abaniel R Bergenstal RM. Prevalence, cost, and burden of diabetic ketoacidosis. Diabetes Technol Ther. (2023) 25:S-75–84. doi: 10.1089/dia.2023.0149 37306442

[45] Green A Bishop RE. Ketoacidosis–where do the protons come from? Trends Biochem Sci. (2019) 44:484–9. doi: 10.1016/j.tibs.2019.01.005 30744927

[46] Soto-Mota A Norwitz NG Clarke K. Why a d-β-hydroxybutyrate monoester? Biochem Soc Trans. (2020) 48:51–9. doi: 10.1042/BST20190240, PMID: 32096539 PMC7065286

[47] Prins PJ Noakes TD Buga A D'Agostino DP Volek JS Buxton JD. Low and high carbohydrate isocaloric diets on performance, fat oxidation, glucose and cardiometabolic health in middle age males. Front Nutr. (2023) 10:1084021. doi: 10.3389/fnut.2023.1084021, PMID: 36845048 PMC9946985

[48] McSwiney FT Wardrop B Hyde PN Lafountain RA Volek JS Doyle L. Keto-adaptation enhances exercise performance and body composition responses to training in endurance athletes. Metabolism. (2018) 81:25–34. doi: 10.1016/j.metabol.2017.10.010 29108901

[49] Volek JS Freidenreich DJ Saenz C Kunces LJ Creighton BC Bartley JM. Metabolic characteristics of keto-adapted ultra-endurance runners. Metabolism. (2016) 65:100–10. doi: 10.1016/j.metabol.2015.10.028 26892521

[50] de Bock M Lobley K Anderson D Davis E Donaghue K Pappas M. Endocrine and metabolic consequences due to restrictive carbohydrate diets in children with type 1 diabetes: an illustrative case series. Pediatr Diabetes. (2018) 19:129–37. doi: 10.1111/pedi.12527 28397413

[51] Nathan DM, Group DER. The diabetes control and complications trial/epidemiology of diabetes interventions and complications study at 30 years: overview. Diabetes Care. (2014) 37:9–16. doi: 10.2337/dc13-2112, PMID: 24356592 PMC3867999

[52] Ranjan A Schmidt S Damm-Frydenberg C Steineck I Clausen TR Holst JJ. Low-carbohydrate diet impairs the effect of glucagon in the treatment of insulin-induced mild hypoglycemia: a randomized crossover study. Diabetes Care. (2017) 40:132–5. doi: 10.2337/dc16-1472 27797928

[53] Volek JS Noakes T Phinney SD. Rethinking fat as a fuel for endurance exercise. Eur J Sport Sci. (2015) 15:13–20. doi: 10.1080/17461391.2014.959564 25275931

[54] Livingstone SJ Levin D Looker HC Lindsay RS Wild SH Joss N. Estimated life expectancy in a Scottish cohort with type 1 diabetes, 2008-2010. JAMA. (2015) 313:37–44. doi: 10.1001/jama.2014.16425 25562264 PMC4426486

[55] Ravnskov U de Lorgeril M Diamond DM Hama R Hamazaki T Hammarskjold B. LDL-C does not cause cardiovascular disease: a comprehensive review of the current literature. Expert Rev Clin Pharmacol. (2018) 11:959–70. doi: 10.1080/17512433.2018.1519391 30198808

[56] Diamond DM O'Neill BJ Volek JS. Low carbohydrate diet: are concerns with saturated fat, lipids, and cardiovascular disease risk justified? Curr Opin Endocrinol Diabetes Obes. (2020) 27:291–300. doi: 10.1097/MED.0000000000000568 32773573

[57] Ravnskov U Diamond DM Hama R Hamazaki T Hammarskjöld B Hynes N. Lack of an association or an inverse association between low-density-lipoprotein cholesterol and mortality in the elderly: a systematic review. BMJ Open. (2016) 6:e010401. doi: 10.1136/bmjopen-2015-010401 PMC4908872 27292972

[58] Hero C Svensson AM Gidlund P Gudbjornsdottir S Eliasson B Eeg-Olofsson K. LDL cholesterol is not a good marker of cardiovascular risk in type 1 diabetes. Diabet Med. (2016) 33:316–23. doi: 10.1111/dme.13007 26498834

[59] Diamond DM Bikman BT Mason P. Statin therapy is not warranted for a person with high LDL-cholesterol on a low-carbohydrate diet. Curr Opinion Endocrinol Diabet Obesity. (2022) 29:497–511. doi: 10.1097/MED.0000000000000764 35938780

[60] Eddy D Schlessinger L Kahn R Peskin B Schiebinger R. Relationship of insulin resistance and related metabolic variables to coronary artery disease: a mathematical analysis. Diabetes Care. (2009) 32:361–6. doi: 10.2337/dc08-0854, PMID: 19017770 PMC2628708

[61] Norwitz NG Feldman D Soto-Mota A Kalayjian T Ludwig DS. Elevated LDL cholesterol with a carbohydrate-restricted diet: evidence for a “lean mass hyper-responder” phenotype. Current developments. Nutrition. (2022) 6:nzab144. doi: 10.1093/cdn/nzab144 PMC8796252 35106434

[62] Yurista SR Chong CR Badimon JJ Kelly DP de Boer RA Westenbrink BD. Therapeutic potential of ketone bodies for patients with cardiovascular disease: JACC state-of-the-art review. J Am Coll Cardiol. (2021) 77:1660–9. doi: 10.1016/j.jacc.2020.12.065 33637354

[63] Soto-Mota A Flores-Jurado Y Norwitz NG Feldman D Pereira MA Danaei G. Increased low-density lipoprotein cholesterol on a low-carbohydrate diet in adults with normal but not high body weight: a meta-analysis. Am J Clin Nutr. (2024) 119:740–7. doi: 10.1016/j.ajcnut.2024.01.009 38237807

[64] Norwitz NG Mindrum MR Giral P Kontush A Soto-Mota A Wood TR. Elevated LDL-cholesterol levels among lean mass hyper-responders on low-carbohydrate ketogenic diets deserve urgent clinical attention and further research. *J Clin Lipidol*. (2022) 16:765–68. doi: 10.1016/j.jacl.2022.10.010 36351849

